# Effects of Exercise on Inflammation and Stress in Older Adults with Varying Chronic Disease Burden

**DOI:** 10.1093/geroni/igaf122.3576

**Published:** 2025-12-31

**Authors:** Emma Plank, Ethan Ouimet, Emily Erlenbach, Stephanie Voss, Edward McAuley, Nicholas Burd, Neha Gothe

**Affiliations:** Northeastern University, Boston, Massachusetts, United States; Northeastern University of Bouve College of Health Sciences, Boston, Massachusetts, United States; EXOS, South San Francisco, California, United States; Mass General Brigham, Boston, Massachusetts, United States; University of Illinois Urbana-Champaign, Urbana, Illinois, United States; University of Illinois Urbana-Champaign, Urbana, Illinois, United States; Northeastern University, Boston, Massachusetts, United States

## Abstract

Regular exercise has been shown to lower both inflammatory and physiological stress markers, such as interleukin-6 (IL-6) and cortisol. Individuals living with chronic diseases (CD) often have higher baseline values of these markers than healthy individuals. The purpose of this study was to examine the effects of 6-month exercise interventions on IL-6 and cortisol in older adults with varying CD burden. Sedentary older adults (n = 142, 64.2 years) participated in 60-min. sessions, 3x/week as a part of an exercise-based randomized controlled trial. IL-6 and cortisol levels were measured at baseline and following the intervention. Participants were categorized into 1 of 3 categories based on health status: healthy (CD0, n = 43), 1 chronic disease (CD1, n = 43), or ≥ 2 chronic diseases (CD2, n = 59). No significant differences were noted on baseline BMI and fitness between CD1 and CD2 however CD0 was significantly different from CD2 (p’s <.02). Baseline BMI was correlated with IL-6 change (r=-.24, p <.05) but not cortisol while baseline fitness was not correlated with either marker. Repeated measures ANOVA demonstrated significant decrease in both IL-6 and cortisol for CD1 (p’s <.01) following the intervention, but not for CD0 and CD2. Participating in three, 60-minute bouts of exercise per week may improve IL-6 and cortisol levels of older adults with 1 CD, thus reducing inflammation and stress associated with CD. Our findings have implications for prevention and CD management, and further research is needed to explore the optimal exercise dosage required to elicit similar reductions for individuals living with a greater CD burden.

